# Dataset on sensitivity of water-energy nexus to Dez Dam power plant operation

**DOI:** 10.1016/j.dib.2020.105454

**Published:** 2020-03-20

**Authors:** Ebrahim Zallaghi, Ali Mohammad Akhoond-Ali, Seyed Mohammad Ashrafi

**Affiliations:** a Faculty of Water Sciences Engineering, Shahid Chamran University of Ahvaz, Ahvaz, Iran; b Department of Hydrology and Water Resources, Faculty of Water Sciences Engineering, Shahid Chamran University of Ahvaz, Ahvaz, Iran; c Department of Civil Engineering, Faculty of Engineering, Shahid Chamran University of Ahvaz, Ahvaz, Iran

**Keywords:** Water-energy nexus (wen), Water-energy productivity (wep), Dez Reservoir Operation

## Abstract

This article explains the time-series data for sensitivity of water-energy nexus to Dez Dam operation located at Dez basin in the southeast of Iran for a period of 54 years (648 months) from October 1964 to September 2018. The utilized data included outflow of the turbine, reservoir inflow, reservoir volume, evaporation, precipitation, spillway, total outflow of the reservoir, elevation, power generation and demand. In this article, operation of Dez Dam is examined for 54 hydrologic years (1964–2018), considering reducing the full supply level (FSL) gradually from its current value to the minimum possible value, increasing the minimum operating level (MOL) gradually to the maximum possible level and operating the Dam at a constant operating level (COL). Also, the concept of water-energy productivity (WEP), defined as the amount of energy produced per unit of water lost in the process, is proposed to measure water-energy nexus (WEN) to changing Dez Dam operation policy. Analysis of the WEN in the context of Dez Dam operation is imperative for improving decision making in the quest for efficient resource use and management.

**Specifications table**

**Table:** 

Subject	Water-energy Nexus for Water Resources Management
Specific subject area	Hydrology and Water Resources Management; Hydropower Energy
Type of data	Table, Figures and Graph
How data were acquired	The dataset was obtained from the Khuzestan Water and Power Authority.
Data format	Raw and analysed data set
Parameters for data collection	• Dez Dam parameters (minimum and maximum turbine outflow, reservoir volume, spillway volume and demand); • The monthly and yearly time-series data of the outflow of the turbine, reservoir inflow, reservoir volume, evaporation, precipitation, spillway, total outflow of the reservoir, elevation, power generation and demand
Description of data collection	Used datasets were provided by Khuzestan Water and Power Authority.
Data source location	Dez Dam located at the Dez basin in Iran.
Data accessibility	All datasets are available in the supplementary file attached to this article.

## Value of the Data

• Data on the outflow of the turbine, reservoir inflow, reservoir volume, evaporation, precipitation, and spillway, total outflow of the reservoir, elevation, power generation and demand in Dez Dam provides an overview of the operation of the dam between 1964 and 2018.
• These datasets can be used to analyse the water resources status and hydropower generated in the Dez reservoir operation.
• The dataset will be beneficial for modelling purposes, relating to Dez Dam.
• The analysis obtained by water-energy productivity (WEP) and water-energy nexus (WEN) can be used by other researchers for comparison.
• Other researchers can employ the WEP and WEN indices in other dams and reservoirs with certainty.

## 1 Data description

In this article, the water-energy productivity (WEP) index is defined in order to perform a suitable surface water resources management. In this regard, new Dez Dam operation policies were suggested herein based on three elevation criteria, namely full supply level (FSL), minimum operating level (MOL), and constant operating level (COL), for the time-series datasets. The time-series dataset of Dez Dam includes outflow of the turbine, reservoir inflow, reservoir volume, evaporation, precipitation, spillway, a total outflow of the reservoir, elevation, power generation and demand for the period of 54 years (1964–2018). The used datasets are shown in Fig. 2. Outflow of the turbine is a volume of outflow from the turbine of Dez Dam (MCM^1^). Reservoir inflow is the volume of inflow to Dez Dam (MCM). Reservoir volume is the storage volume of Dez Dam (MCM). Evaporation is the depth of evaporation from the area of Dez Dam (mm). Precipitation is the depth of precipitation in the area of the Dez Dam (mm^2^). The spillway is the volume of overflow from Dez Dam (MCM). Total outflow of the reservoir is a volume of outflow from Dez Dam (MCM). Elevation is the Dez reservoir water height above sea level (MASL^3^). Power generation is generating electric power from the Dez power plant (Mwh^4^). Demand is defined as the volume of downstream water demand of Dez Dam (MCM) to supply municipal, agricultural and industrial consumptions.

Fig. 3, Fig. 4, Fig. 5 show the average water-energy productivity (WEP) under varied Dez Dam operation scenarios in drought, wet and normal periods, respectively. Table 1 displays the analysis of performance criteria for Dez reservoir operation including time-based and volumetric reliability, resilience, vulnerability and sustainability indices.

**Table 1:** Analysis of performance criteria for Dez reservoir operation (T-reliability, V-reliability, resilience, vulnerability and sustainability indices).

Year	T-Rel	Res	Vul	Sus	V-Rel	Year	T-Rel	Res	Vul	Sus	V-Rel
**1964–1965**	66.67	33.33	25.42	54.93	93.09	**1991–1992**	91.67	66.67	18.61	79.23	97.88
**1965–1966**	75.00	50.00	20.07	66.92	93.93	**1992–1993**	100.00	NaN	0.00	NaN	100.00
**1966–1967**	16.67	10.00	50.90	20.15	72.87	**1993–1994**	83.33	50.00	17.17	70.14	96.79
**1967–1968**	41.67	11.11	37.37	30.72	85.15	**1994–1995**	83.33	50.00	5.51	73.29	98.80
**1968–1969**	75.00	25.00	24.69	52.07	95.79	**1995–1996**	75.00	66.67	12.35	75.96	96.95
**1969–1970**	41.67	11.11	50.56	28.39	74.71	**1996–1997**	50.00	33.33	44.33	45.27	85.30
**1970–1971**	41.67	25.00	42.88	39.04	83.77	**1997–1998**	83.33	50.00	12.17	71.53	97.51
**1971–1972**	75.00	66.67	13.45	75.64	97.08	**1998–1999**	33.33	0.00	56.26	0.00	81.99
**1972–1973**	75.00	25.00	27.75	51.36	93.84	**1999–2000**	8.33	0.00	57.86	0.00	69.68
**1973–1974**	50.00	28.57	44.36	43.00	89.33	**2000–2001**	25.00	22.22	59.66	28.19	74.48
**1974–1975**	66.67	60.00	14.52	69.93	94.56	**2001–2002**	75.00	75.00	11.66	79.21	96.89
**1975–1976**	83.33	50.00	24.62	67.97	95.82	**2002–2003**	66.67	60.00	8.09	71.64	97.63
**1976–1977**	66.67	20.00	57.13	38.52	84.42	**2003–2004**	66.67	50.00	11.85	66.48	95.78
**1977–1978**	58.33	40.00	57.45	46.30	80.19	**2004–2005**	83.33	50.00	17.49	70.05	96.25
**1978–1979**	66.67	25.00	21.78	50.70	94.13	**2005–2006**	83.33	25.00	17.61	55.58	96.20
**1979–1980**	75.00	50.00	21.15	66.62	95.38	**2006–2007**	91.67	50.00	14.09	73.29	98.35
**1980–1981**	75.00	40.00	32.83	58.63	94.64	**2007–2008**	33.33	25.00	79.37	25.81	65.28
**1981–1982**	83.33	25.00	19.11	55.24	96.97	**2008–2009**	0.00	0.00	69.64	0.00	48.63
**1982–1983**	100.00	66.67	4.44	86.05	99.07	**2009–2010**	50.00	14.29	70.72	27.55	81.22
**1983–1984**	33.33	0.00	47.41	0.00	79.75	**2010–2011**	25.00	0.00	71.79	0.00	79.50
**1984–1985**	66.67	75.00	15.42	75.06	95.43	**2011–2012**	8.33	9.09	52.71	15.30	64.04
**1985–1986**	66.67	50.00	24.22	63.21	94.97	**2012–2013**	8.33	0.00	70.19	0.00	70.54
**1986–1987**	100.00	100.00	4.71	98.41	99.46	**2013–2014**	25.00	10.00	51.82	22.92	79.95
**1987–1988**	100.00	NaN	0.00	NaN	100.00	**2014–2015**	33.33	22.22	41.38	35.15	83.76
**1988–1989**	83.33	66.67	14.50	78.02	97.46	**2015–2016**	58.33	40.00	29.66	54.75	92.44
**1989–1990**	83.33	50.00	6.29	73.09	98.64	**2016–2017**	66.67	33.33	19.92	56.25	94.07
**1990–1991**	33.33	12.50	60.02	25.54	82.86	**2017–2018**	0.00	0.00	65.49	0.00	54.42

## 2 Experimental design, materials, and methods

**Fig. 1:**
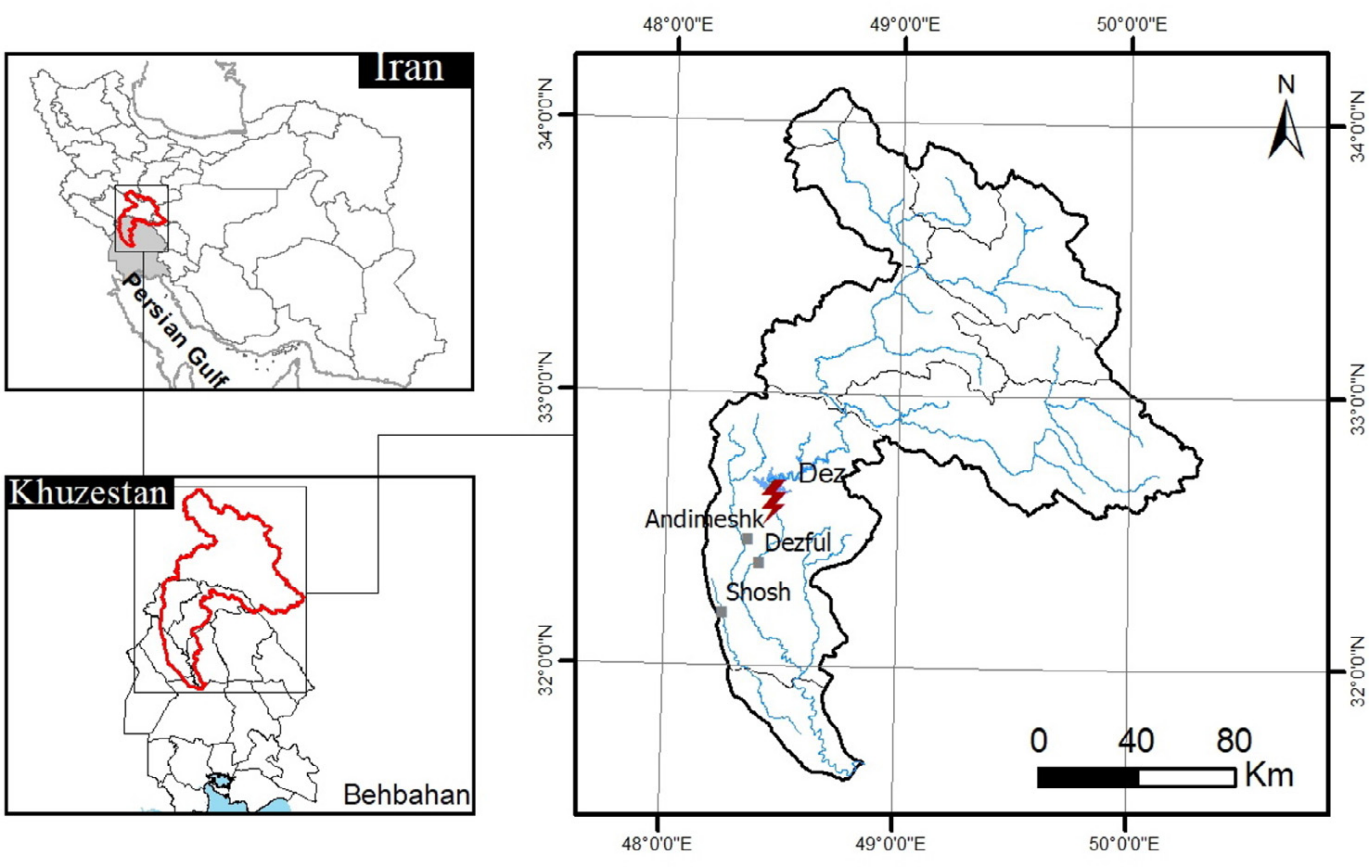
Location of the Dez Dam.

The Dez reservoir is located in the Zagros Mountains in the southwest Iran and was created by the construction in 1963 of Dez Dam with a height of 203 m. An underground power house contains eight 65 MW^5^ units for a total installed capacity of 520 MW, which generates the average of 2400 GWh/year^6^ energy over an operating period of 45 years. The minimum and maximum water levels of the reservoir operation are 300 m and 352 m from sea level, respectively. Flow releases are through the spillway, power tunnels and three low-level irrigation outlets. The original reservoir volume is 3315 MCM and, over an operating period of 40 years, the storage volume has been reduced to 2600 MCM by sedimentation. Fig. 1 illustrates the lake of Dez reservoirs within the Dez river basin.

**Fig. 2:**
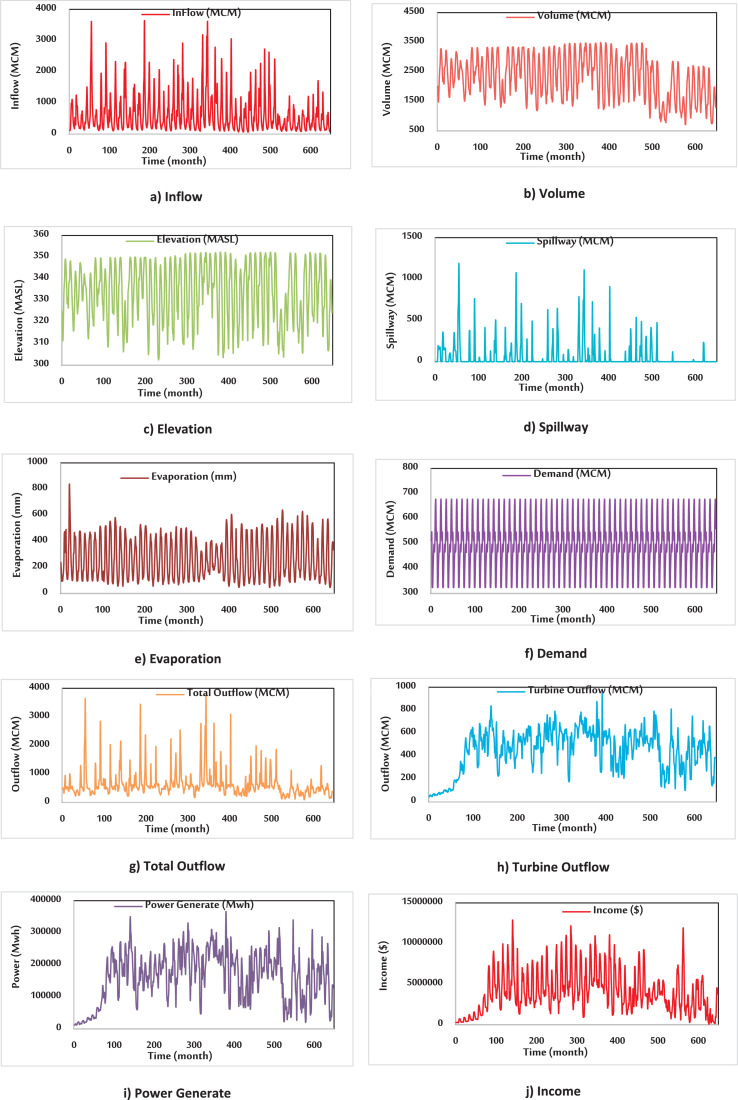
Time-series diagram of the Dez Dam datasets (a) Inflow (MCM), (b) Volume (MCM), (c) Elevation (MASL), (d) Spillway (MCM), (e) Evaporation (mm), (f) Demand (MCM), (g) Total outflow (MCM), (h) Turbine outflow (MCM), (i) Power generation (Mwh), (j) Income ($).

In this article, at first, the years of operation were divided into three categories based on the SPI^7^ drought index, wet, drought and normal. Then, for the time-series datasets (1964–2018), three elevation criteria were suggested based on reservoir elevation, namely full supply level (FSL), minimum operating level (MOL) and constant operating level (COL). Also, the water-energy produced (WEP) was proposed to measure the sensitivity of the water-energy nexus (WEN) of the Dez Dam operation policy. The water-energy productivity (WEP) concept is defined as the amount of energy produced per unit of water lost in the process [1]. Accordingly, the lower the WEP, the more the water lost for producing one unit of energy. The water-energy productivity (WEP) indicator is defined as the following equation:

(1) $$WEP\left(Gwh/BCM\right)=\frac{Ep}{Wl}$$

where WEP is the water-energy productivity indicator for Dez Dam, Ep is the energy production of Dez Dam, which is expressed in Gigawatt hour (Gwh), and Wl is the water losses of Dez Dam, which is expressed in billion cubic meters (BCM^8^) [1], [2], [3], [4].

## 3 Reservoirs performance criteria

Performance criteria are used to evaluate water management policies and enable the comparison of alternative policies. Probability-based performance criteria include time-based and volumetric reliability, resilience, vulnerability and sustainability indices [5], [6].

(2) $$\text{Rel}=\left(1-\frac{\text{ND}e_{f}}{T}\right)\times \;100,\;\;\text{ND}e_{f}=\text{No}.\;\text{of}\;\text{times}\left(De_{t}>Re_{t}\right)$$

(3) $$\alpha_{v}=\frac{Re_{\text{Total}}}{De_{Total}}\times \;100$$

(4) $$\text{Val}=\text{max}\left\{\frac{\left(De_{t}-Re_{t}\right)}{De_{t}}\right\}\times \;100,\;t=1,2,\ldots ,T$$

(5) $$\text{Res}=\frac{\begin{array}{c} T\\ N\\ t=1 \end{array}\left(\text{De}f_{t+1}=0\;|\;\text{De}f_{t}\rangle 0\right)}{\begin{array}{c} T\\ N\\ t=1 \end{array}\left(\text{De}f_{t}>0\right)}\times \;100,\;t=1,2,\ldots ,T$$

(6) $$SI^{i}={\left\{\text{Re}l^{i}\times \text{Re}s^{i}\times \;\left(1-\text{Vu}l^{i}\right)\right\}}^{1\;/\;3}$$

where Rel is the time-based reliability index, a_v_ is the volumetric reliability index, Val-is the vulnerability index, Res is the resilience index, SI is the sustainability index [5], De_t_ is the demand of Dez reservoir in period t, Re_t_ is the release from Dez reservoir in period t, NDe_f_ is the number of times De_t_>Re_t_ and Def_t_ is the deficit of Dez Dam downstream demand in period t.

**Fig. 3:**
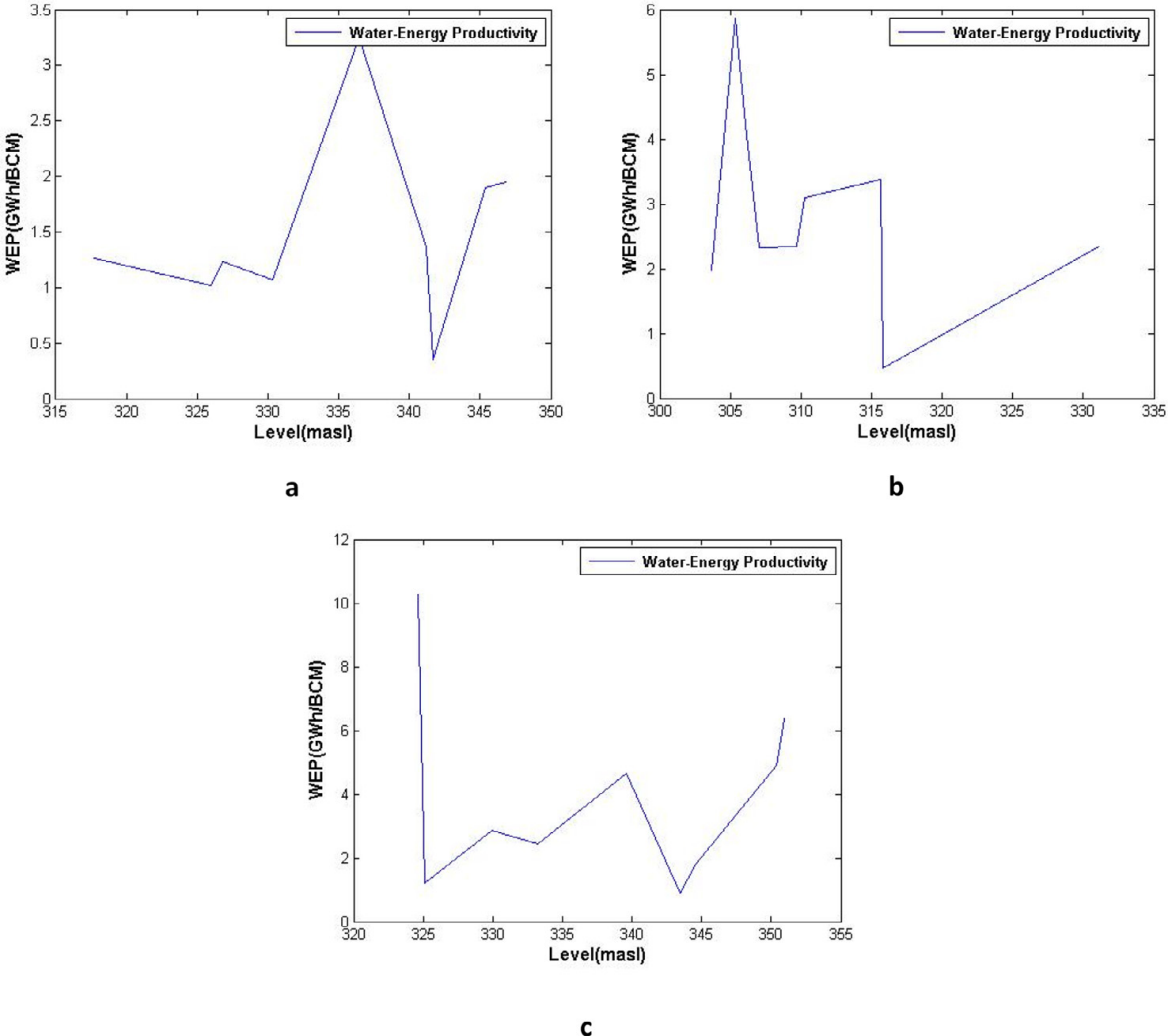
Average water-energy productivity (WEP) under varied Dez Dam operation scenarios, (a) Full supply level (FSL), (b) Minimum operating level (MOL), and (c) Constant operating level (COL), in drought periods.

**Fig. 4:**
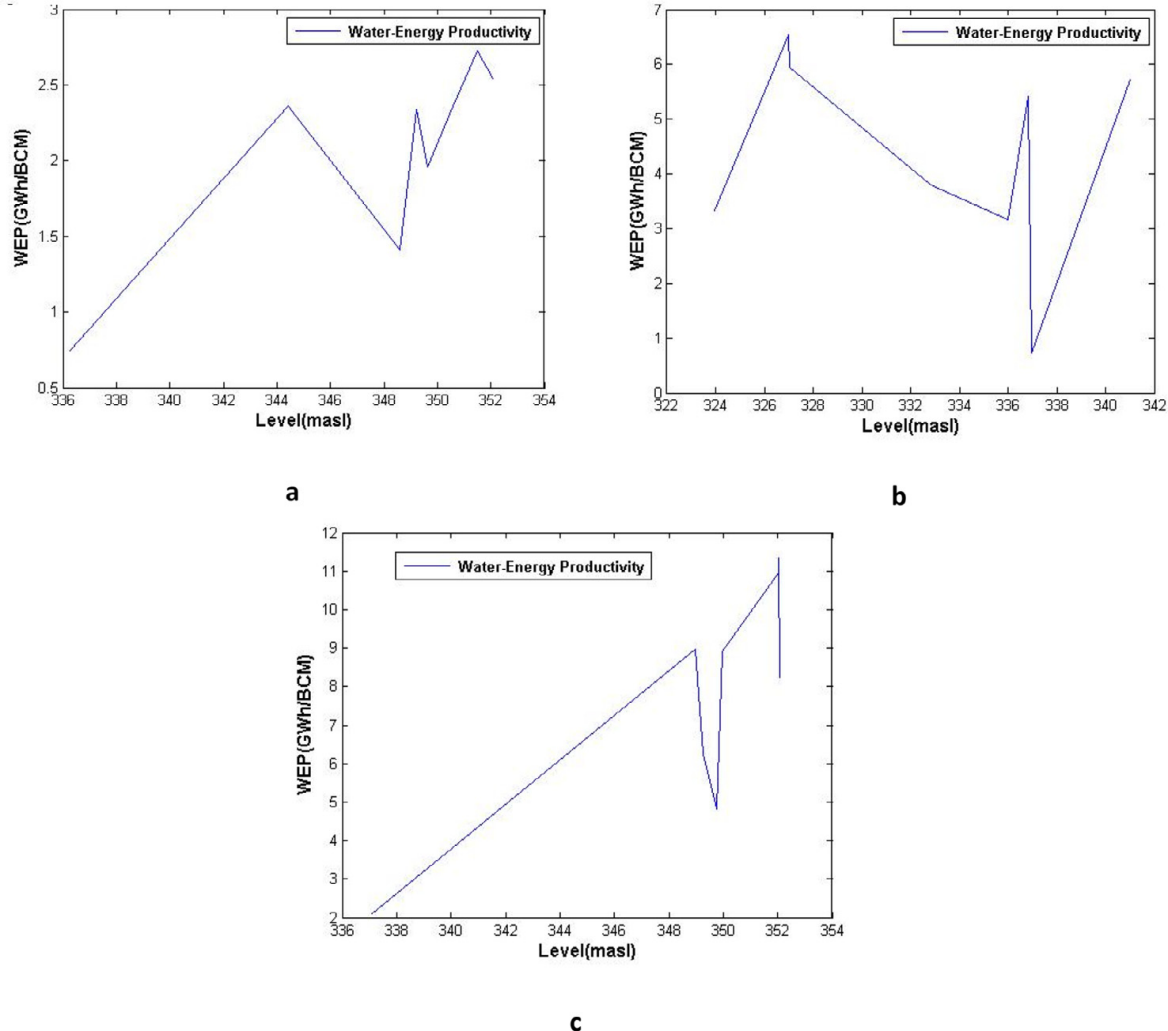
Average water-energy productivity (WEP) under varied Dez Dam operation scenarios, (a) Full supply level (FSL), (b) Minimum operating level (MOL), and (c) Constant operating level (COL), in wet periods.

**Fig. 5:**
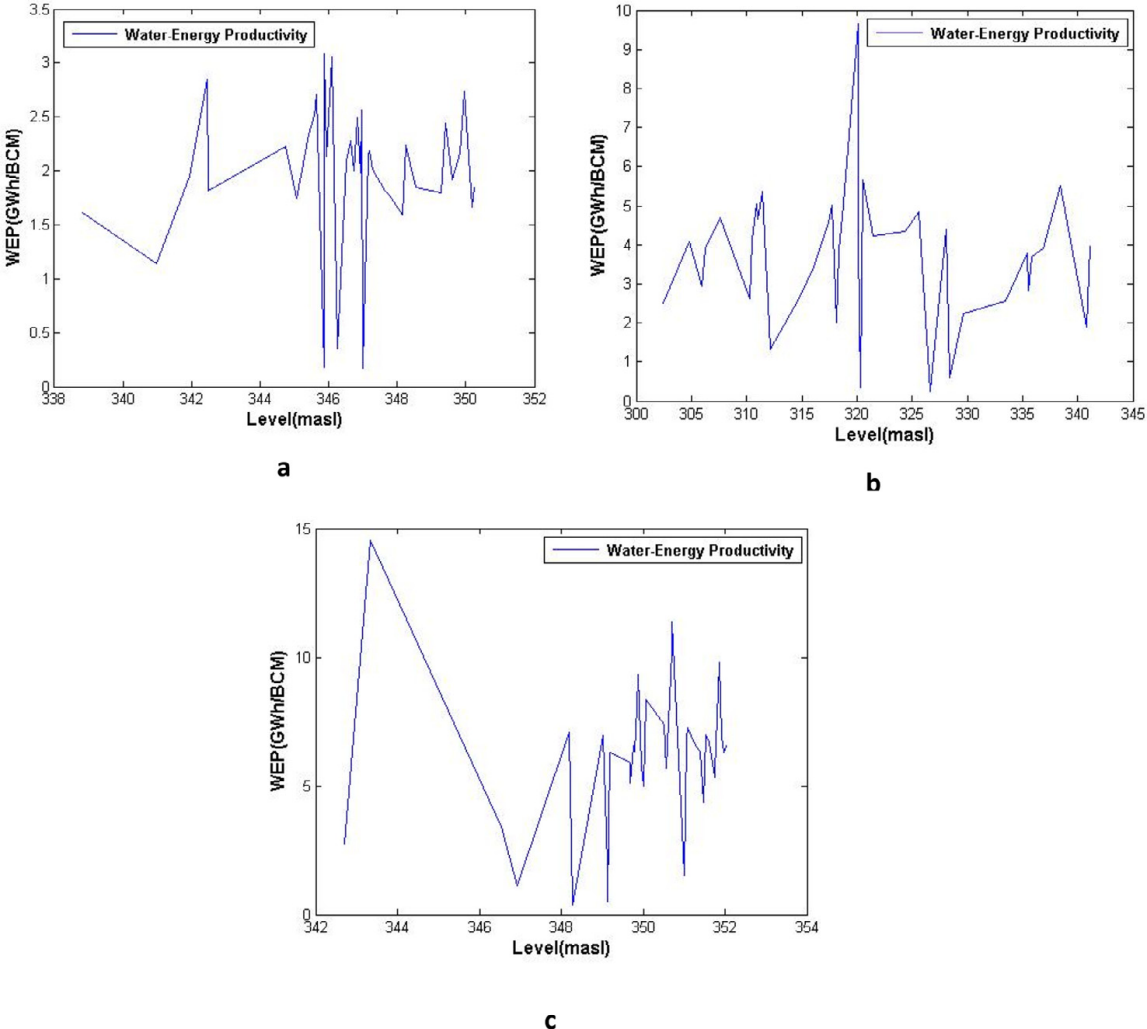
Average water-energy productivity (WEP) under varied Dez Dam operation scenarios, (a) Full supply level (FSL), (b) Minimum operating level (MOL), and (c) Constant operating level (COL), in normal periods.

All analyses of this data article for water-energy nexus are presented in Fig. 3, Fig. 4, Fig. 5.

## Declaration of Competing Interest

The authors declare that they have no known competing financial interests or personal relationships which have, or could be perceived to have, influenced the work reported in this article.

## Appendix Supplementary materials

Image, application 1

Image, application 2

## Footnotes

1 Million Cubic Meters.

2 millimeter.

3 Meters Above Sea Level.

4 Megawatt-hour.

5 Megawatts.

6 Gigawatt hours per year.

7 Standard Precipitation Index.

8 Billion Cubic Meters.
